# Solid Pseudopapillary Tumor of the Pancreas: An Unusual Cause of Abdominal Pain

**DOI:** 10.7759/cureus.1252

**Published:** 2017-05-16

**Authors:** Talal El Imad, Fady G. Haddad, Mayurathan Kesavan, Liliane Deeb, Sherif Andrawes

**Affiliations:** 1 Department of Internal Medicine, Staten Island University Hospital; 2 Department of Gastroenterology, Staten Island University Hospital

**Keywords:** pseudopapillary tumor, immunohistochemical stains, abdominal pain

## Abstract

Solid-pseudopapillary neoplasm (SPN) of the pancreas is a rare tumor that accounts for less than one percent of pancreatic tumors. The diagnosis could be challenging as SPN tend to manifest with nonspecific abdominal symptoms, variable radiological features, and inconsistent morphology. The cellular origin of SPN is unclear and might involve ductal, acinar and endocrine stem cells.

We report a rare case of a 27-year-old female who presented with intermittent abdominal pain for two years, associated with a decrease in appetite. Her medical history was significant for abdominoplasty five years ago. Vital signs were stable. Physical examination revealed mild epigastric tenderness. Laboratory tests were unremarkable. Contrast computed tomography (CT) scan of the abdomen showed a 2 x 2 cm indeterminate pancreatic tail lesion. An endoscopic ultrasound (EUS) disclosed a 2.1 x 1.8 cm hypoechoic mass in the tail of the pancreas.Trans-gastric fine needle aspiration was obtained to show clusters of uniform neoplastic cells with abundant cytoplasm and oval bean-shaped nuclei. Immunohistochemical stains were positive for beta-catenin, Vimentin, CD10, CD56, cytokeratin-7 (Ck7), Cyclin D1, and negative for chromogranin, epithelial-cadherin (E cadherin) which was consistent with a pseudopapillary tumor. The patient underwent a robotic assisted en-bloc distal pancreatectomy and splenectomy. There were no intra-abdominal metastases.

SPN is a rare tumor characterized by a specific immunohistological pattern which makes it highly distinct from other pancreatic neoplasms particularly neuroendocrine tumors, acinar carcinomas, and carcinoids. It is important to differentiate SPN from other pancreatic neoplasms because it is characterized as low potential for malignancy and a favorable prognosis after resection, with a five-year survival rate approaching 85%-95%.

## Introduction

Solid-pseudopapillary neoplasm (SPN) of the pancreas also known as Frantz’s tumor is an uncommon tumor that mainly occurs in females in their second to fourth decades of life [1]. It accounts for less than one percent of pancreatic tumors and typically arises from the pancreatic tail [2]. Establishing the diagnosis can be difficult owing to the non-specific clinical presentation as well as highly variable radiological and pathological features. Informed consent statement was obtained for this study.

## Case presentation

A 27-year-old female presented to our emergency department (ED) with abdominal pain. She describes a two-year history of intermittent epigastric pain, dull, none radiating and not related to food intake associated with the decrease in her appetite. Her medical history was significant for abdominoplasty five years ago in the Dominican Republic. She denies taking any medications, drinking alcohol or smoking cigarettes. The patient underwent an upper endoscopy one month prior to presentation as part of an outpatient workup of her abdominal pain that was completely normal. Upon presentation, the patient was afebrile and vital signs showed a blood pressure of 126/78, heart rate of 76. Physical examination revealed mild epigastric tenderness whereas the rest of her examinations were normal. Laboratory tests were overall unremarkable (Table 1).

**Table 1:** Laboratory test results

	Hemoglobin Level	Leukocyte Count	Alanine Transaminase	Aspartate Transaminase	Alkaline Phosphate	Lipase	Total Bilirubin
Results	14.2 g/dl	9000/ml	34 IU/L	41 IU/L	56 IU/L	26 IU/L	0.7 mg/dl

**Figure 1:**
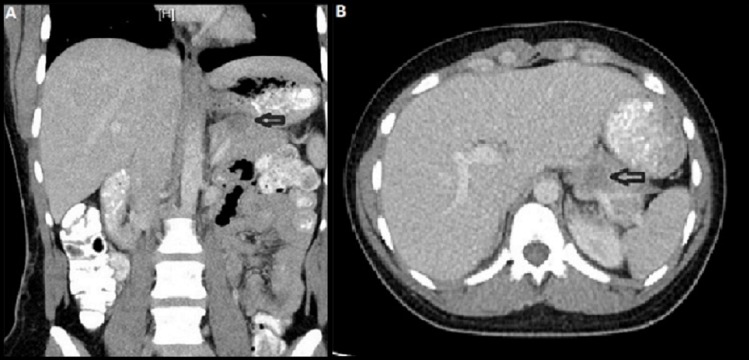
Computed tomography (CT) scan of the abdomen (A): Coronal view of abdominal CT scan showing a 2 x 2 cm pancreatic lesion (arrow); (B): Axial view of abdominal CT scan showing the pancreatic lesion (arrow)

Contrast computed tomography (CT) scan of the abdomen was done in the ED and showed a 2 x 2 cm indeterminate pancreatic tail lesion (Figure 1).

**Figure 2:**
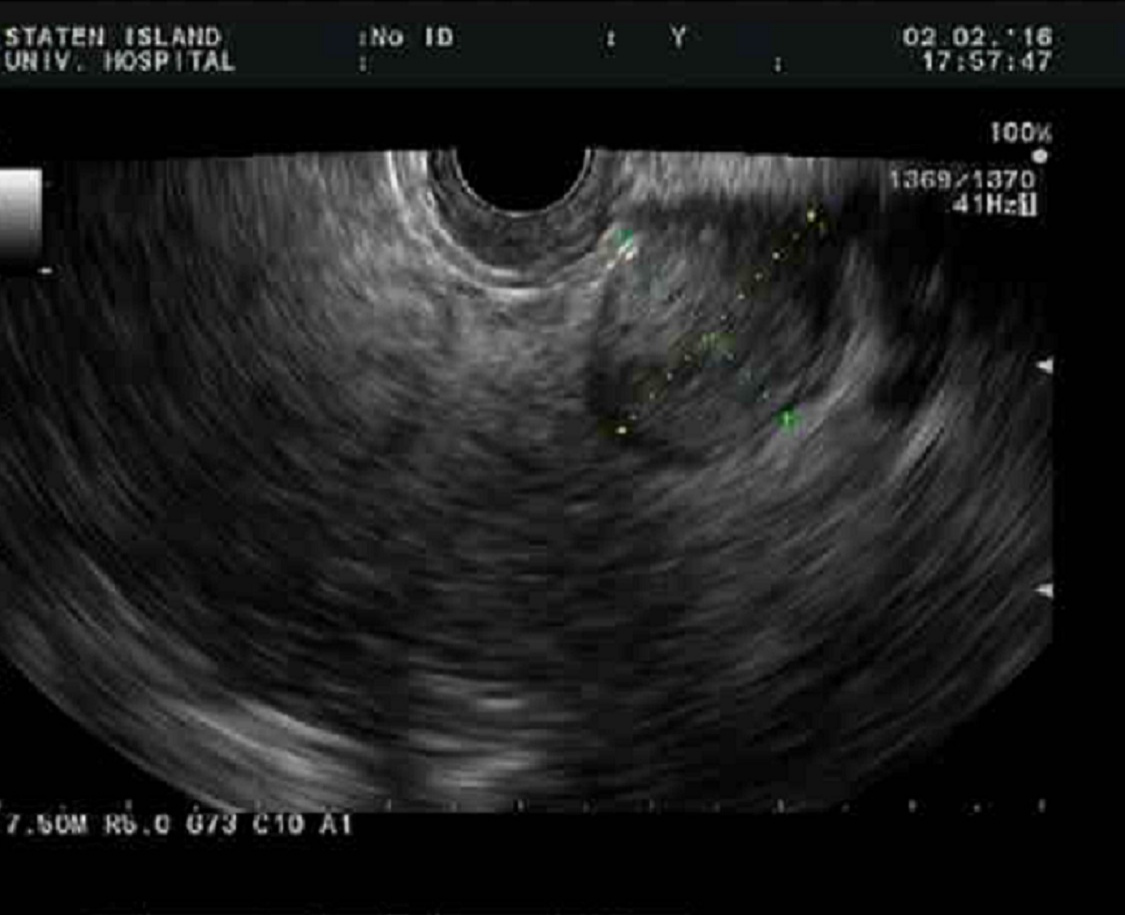
Endoscopic ultrasound Endoscopic ultrasound image showing 2.1 x 1.8 cm hypoechoic pancreatic tail mass

For further evaluation of the lesion, an endoscopic ultrasound (EUS) was performed which disclosed a 2.1 x 1.8 cm hypoechoic mass in the tail of the pancreas (Figure 2).

**Figure 3:**
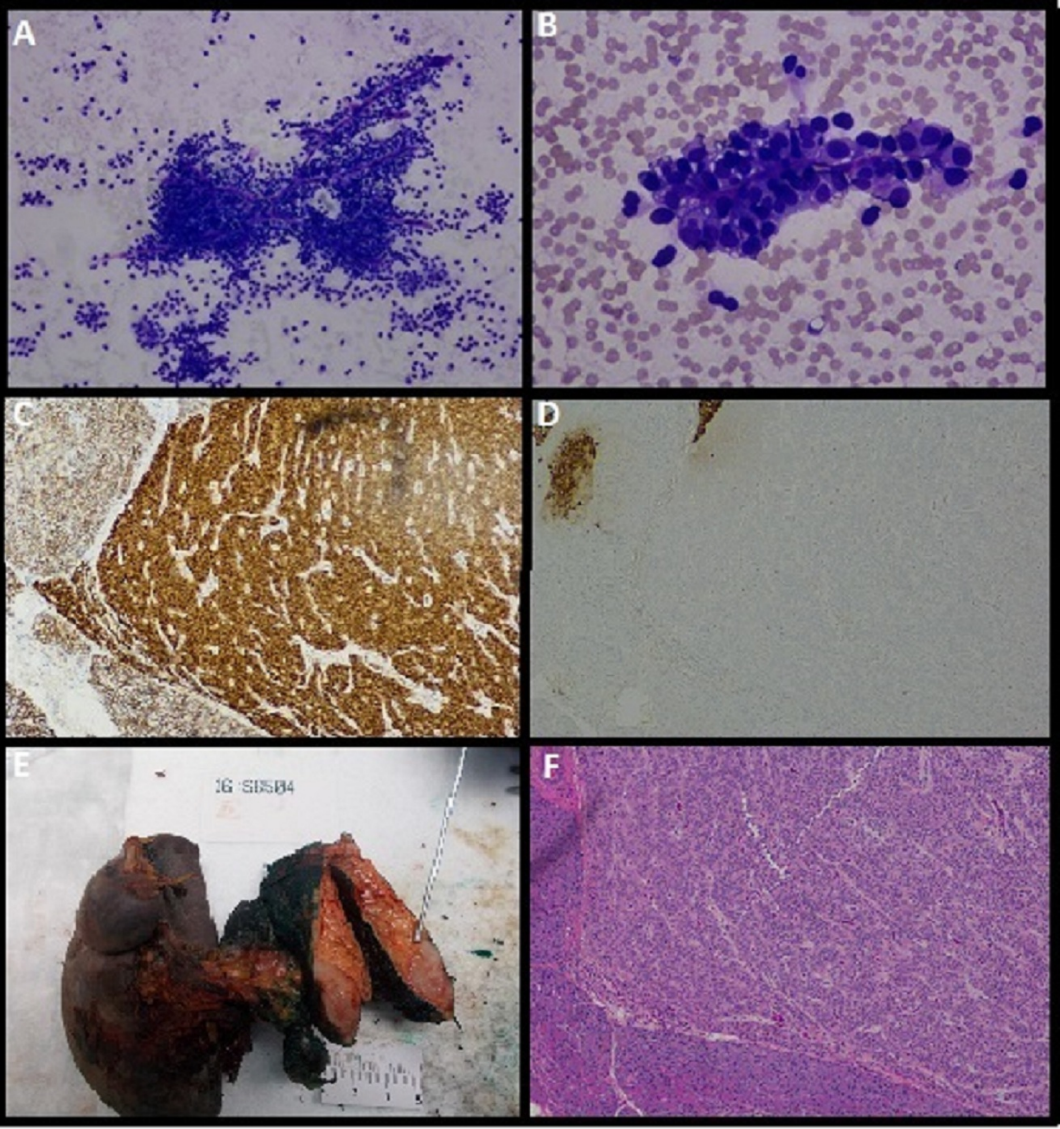
Pathology (A and B): Cytology showing neoplastic cells containing finely vacuolated cytoplasm and oval bean shaped nuclei lining hyalinized vascular stalk; (C): Pancreatic tumor cells with positive nuclear staining for beta catenin; (D): Pancreatic tumor cells with negative staining for chromogranin; (E): Gross pathology specimen of the tumor; (F): Histology specimen showing encapsulated neoplastic cells surrounded by normal pancreatic tissue

Trans-gastric fine needle aspiration was obtained revealing clusters of uniform neoplastic cells with abundant cytoplasm and oval bean-shaped nuclei. Immunohistochemical stains were positive for beta-catenin, Vimentin, CD10, CD56, Ck7, Cyclin D1, and negative for chromogranin, E-Cadherin (Figure 3). These findings were consistent with a pseudopapillary tumor.

The patient underwent a robotic assisted en-bloc distal pancreatectomy and splenectomy. There were no intra-abdominal metastases. The patient had an uneventful post-operative recovery and upon further follow-up reported being pain-free.

## Discussion

Solid pseudopapillary neoplasms were first described in 1959 in a series of three cases by Frantz. Ten years later, Hamoudi, et al. reported the characteristic features of pancreatic SPNs on electron microscopy [3]. These rare tumors constitute around one to two percent of all pancreatic neoplasms [1-2], but with the advancements in imaging and procedural modalities, they are becoming increasingly reported. In one of the largest case series, Papavradimis, et al. described a tenfold increased prevalence among females with a mean age of 22 years [1].

Pancreatic SPNs tend to be slowly growing tumors and the majority of patients are asymptomatic [4]. When symptomatic, the most commonly reported complaint is the diffuse nonspecific abdominal pain [5]. Vomiting and early satiety develop later in the course of the disease with enlargement of the tumor causing a mass effect. Our patient had intermittent, vague abdominal pain with anorexia for almost two years before the tumor was detected.

The initial diagnosis of pancreatic SPNs relies mostly on imaging as there are no specific tumor markers for this entity. CT scan has a good sensitivity rate in detecting these tumors [6]. The presence of an SPN is highly suggested when certain pathognomonic features are identified on CT scan [7]: well-defined, encapsulated mass with areas of central calcification, necrosis or hemorrhage. In contrast to other pancreatic tumors, SPNs tend to have similar peripheral enhancement with the surrounding pancreatic parenchyma during both arterial and venous phases. Some experts recommend using magnetic resonance imaging (MRI) as these tumors have some characteristic properties [8] that can differentiate them from other pancreatic tumors: heterogeneous high signal intensity on T2 and an early peripheral heterogeneous enhancement on dynamic imaging.

Once the diagnosis is suggested by imaging, preoperative histological identification can be done by fine needle aspiration with up to 70% sensitivity and specificity [4-5]. The retrieved cells are usually ovoid or polygonal in shape with typical small central nuclei and abundant cytoplasm [9]. In fact, more than 90% of these tumors stain positive for Vimentin, enolase, alpha1 antitrypsin, alpha1 antichymotrypsin [10], beta-catenin and negative for E-cadherin, chromogranin, and CK19. In our case, fine needle aspiration revealed cells with abundant cytoplasm and oval bean-shaped nuclei, staining positive for beta-catenin, Vimentin, CD10, CD56, Ck7, Cyclin D1, and negative for chromogranin, E-Cadherin.

The mainstay of therapy is surgical resection. En bloc resection of the tumor with clear margins provides a high cure rate with an excellent overall survival. The presence of lymphovascular or capsular invasion, local extension, lymph node involvement and liver metastases is associated with a poor outcome. Nevertheless, the long-term survival remains high compared to adenocarcinomas with similar features and thus surgical resection is still recommended for SPNs with poor prognostic features.

The use of chemotherapy in SPNs is not well studied. Some case series suggested administering systemic chemotherapy for patients with poor prognostic features or metastatic disease but the evidence behind this recommendation is lacking. Certain reports described using gemcitabine prior to surgical resection to achieve shrinkage of the tumor size.

## Conclusions

Despite the increasing number of SPNs detected incidentally by imaging, these tumors remain rare and the presentation of individual cases can be unique and challenging. Hence, it is essential to keep a high index of suspicion for this entity when treating a young female with non-specific abdominal pain and evidence of a pancreatic lesion. It is crucial to distinguish SPNs from other pancreatic tumors because these neoplasms have a high cure rate and excellent long-term survival following surgical resection even in the presence of poor prognosticators. 
